# The Effects and Mechanisms of Ti-Fu-Kang Decoction in Alleviating Central Fatigue: Insights from Network Pharmacology and Metabolomics

**DOI:** 10.3390/ph18101545

**Published:** 2025-10-14

**Authors:** Yifei Zhang, Zehan Zhang, Qingqian Yu, Qinghuan Shi, Bijuan Lan, Yan Liu, Weiyue Zhang, Feng Li

**Affiliations:** 1 School of Traditional Chinese Medicine, Beijing University of Chinese Medicine, Beijing 102488, China; jinqian0326@163.com (Y.Z.); zhangzehan2385@sina.com (Z.Z.); yuqqzy@163.com (Q.Y.); shegnfan@163.com (Q.S.); lanbijuan0523@163.com (B.L.); liuy806@163.com (Y.L.); 2 School of Nursing, Beijing University of Chinese Medicine, Beijing 102488, China

**Keywords:** Ti-Fu-Kang, central fatigue, network pharmacology, metabolomics, rats

## Abstract

**Background:** Although Ti-Fu-Kang (TFK) decoction has been clinically used for fatigue management, the systematic understanding of its mechanisms, particularly against central fatigue, remains largely unknown. This study is the first to employ an integrative approach of network pharmacology and metabolomics to explore the mechanisms of TFK against central fatigue. **Methods:** The central fatigue rat model was established using the modified multiple platform method in conjunction with alternate-day fasting. Behavioral alterations were evaluated through six behavioral tests, while brain injury was assessed through HE and Nissl staining. Serum metabolic indicators were analyzed to identify fatigue-related metabolic disturbances. Western blot analysis was used to assess the protein phosphorylation level of PI3K and AKT1. Oxidative stress was assessed by measuring superoxide dismutase, malondialdehyde, and glutathione peroxidase activities. Network pharmacology and serum metabolomics investigated the molecular mechanisms and metabolic pathways. **Results:** TFK significantly ameliorated behavioral abnormalities and brain pathological damage in central fatigue model rats. Network pharmacology analysis and in vivo experiment revealed that TFK may mediate biological processes such as oxidative stress and neuron death via the PI3K-AKT signaling pathway. Moreover, analysis of serum fatigue-related metabolic indicators indicated that TFK significantly modulated metabolic disruptions by elevating the levels of glucose, liver glycogen, and muscle glycogen and reducing the levels of alanine aminotransferase, aspartate aminotransferase, blood urea nitrogen, creatine kinase, lactate, and lactate dehydrogenase in central fatigue rats. Serum metabolomics analysis revealed that TFK ameliorates central fatigue by modulating amino acid metabolism, specifically by altering the levels of leucine and L-tryptophan, which subsequently contributes to the restoration of 5-hydroxytryptamine and dopamine homeostasis. **Conclusions:** This study elucidates the potential therapeutic mechanism of TKF in alleviating central fatigue, providing a scientific and theoretical basis for broader application and development of TFK.

## 1. Introduction

Fatigue is a common health issue, with a high incidence rate [1]. Long-term fatigue can lead to reduced productivity among workers, deterioration in health, and is a major contributor to the morbidity and mortality rates in the workplace [2,3]. Currently, research progress in the treatment of fatigue remains quite limited, with a lack of targeted and effective therapeutic interventions and interventions [4,5,6].

Fatigue can be classified into central fatigue and peripheral fatigue based on differences in pathophysiological mechanisms [7]. Central fatigue is a form of neuro-muscular dysfunction resulting from impairments within the central nervous system, manifesting as loss of mental drive, decreased appetite, increased somnolence, and reduced psychological vigilance [7,8,9]. Dysbiosis and perturbed metabolomics may serve as potential factors contributing to fatigue [10]. The levels of amino acids in the serum, particularly the levels of neuroactive tryptophan metabolites, play a crucial role in the development of central fatigue [11]. Therefore, modulating the levels of fatigue-related metabolites in the serum emerges as a potential therapeutic target for central fatigue.

Ti-Fu-Kang (TFK) is a widely used decoction of traditional Chinese medicine (TCM) in clinical practice, consisting of five TCM herbs: Huang Qi (*Radix Astragali*), Zhi Qiao (*Aurantii Fructus*), Shan Zha (*Fructus Aurantii*), Chai Hu (*Bupleuri Radix*), and Dang Gui (*Radix Angelicae Sinensis*). TFK has long been employed in clinical practice for fatigue management, with demonstrated efficacy in alleviating fatigue symptoms [12]. Current research also demonstrate that TFK exhibits significant therapeutic effects on rat models of physical fatigue as well as psychological fatigue [13,14]. Based on previous studies, TFK has been included in the Chinese Scientific and Technological Achievements Registration System (Number: 39430140). Moreover, the modified formula of TFK has also been shown to significantly modulate cognitive and emotional disturbances in central fatigue rats [15]. Additionally, unique bioactive compounds present in the constituent herbs of TFK, including Astragalus polysaccharides, Astragaloside from Huang Qi, and the polysaccharide BCP-2 from Chai Hu, have all been reported to exhibit notable anti-fatigue activity, suggesting their potential utility in managing chronic fatigue [16,17,18]. However, how these specific components collectively contribute to TFK’s anti-central fatigue effects through a systems-level mechanism has not been investigated.

Network pharmacology has gained widespread application in research on TCM compound prescriptions [19]. Network pharmacology effectively integrates TCM with modern medicine, facilitating a deeper understanding of the molecular mechanisms of the TCM compound preparations [20]. Metabolomics is an emerging field that involves the comprehensive analysis of the dynamic metabolic profiles in living systems, aiming to discover new targets for the TCM compound preparations at the level of the biological metabolism network and elucidate their overall mechanisms [21,22]. This study aims to elucidate the therapeutic effects of TFK on central fatigue through in vivo experiments and systematically explore the mechanisms of TFK through the combination of network pharmacology and metabolomics.

## 2. Results

### 2.1. The Results of Ultra High-Performance Liquid Chromatography-Mass Spectrometry (UHPLC-MS/MS) Analysis

**Figure 1:**
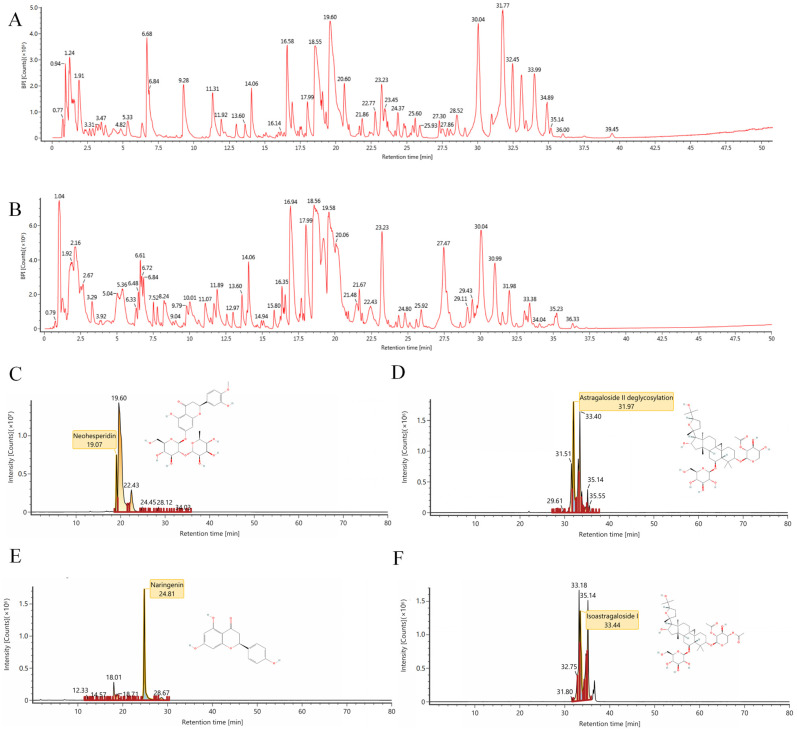
Ion chromatography of bioactive compounds in TFK. Total ion chromatogram monitored in positive (**A**) and negative (**B**) ion modes for TFK. (**C**–**F**) The representative chemical components identified in TFK including neohesperidin (**C**), astragaloside II (**D**), naringenin (**E**), and isoastragaloside I (**F**).

Through UHPLC-MS/MS analysis, a total of 210 compounds were identified from the TKF sample (Figure 1 and Supplementary Tables S1 and S2), with 62 compounds for which compound names could not be obtained through database retrieval. Following the screening criterion of Oral Bioavailability (OB) ≥ 30% and Drug-Likeness (DL) ≥ 0.18, a total of 30 active compounds were obtained (Supplementary Table S3).

### 2.2. Network Pharmacology-Based Analysis

#### 2.2.1. Potential Therapeutic Targets of TFK in the Treatment of CF

**Figure 2:**
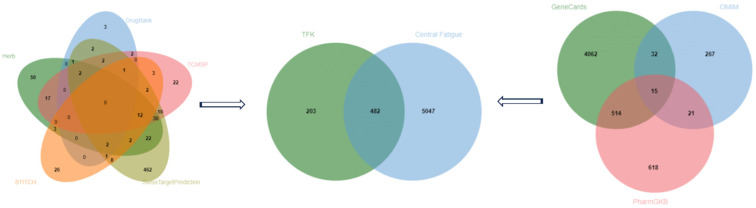
Venn diagram. The Venn diagram on the left illustrates the number of TFK-related targets retrieved from various databases, while the Venn diagram on the right displays the number of central fatigue-related targets retrieved from different databases. In the middle Venn diagram, the common targets between TFK and central fatigue, as well as the unique targets for each, are depicted.

After eliminating duplicates, a total of 685 TFK related targets were discovered from the five databases (Figure 2). A total of 5529 central fatigue related targets were retrieved from the GeneCards, OMIM and PharmGKB databases. Moreover, 482 common targets of TFK and central fatigue were identified as potential therapeutic candidates (Figure 2 and Supplementary Table S4).

#### 2.2.2. Protein–Protein Interaction (PPI) Network Construction and Topological Screening

**Figure 3:**
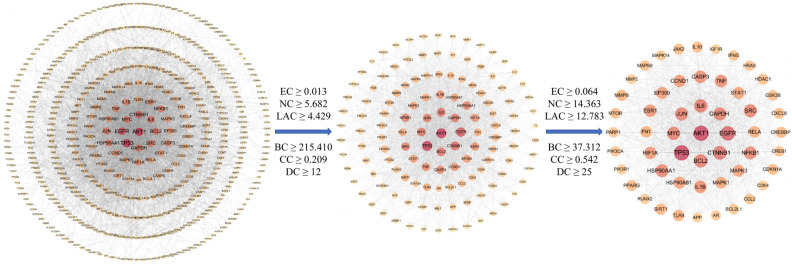
PPI network and hub targets screening. The upmost figure was the PPI network of 482 common targets between TFK and central fatigue. From left to right shows the topological screening process of the hub targets. The BC, CC, DC, EC, LAC, and NC values of each target were computed, and nodes with values greater than or equal to the median were identified as hub targets.

The STRING database was used to gather the protein–protein interaction information amongst the therapeutic targets of TFK and build the PPI network. With a minimum interaction score of 0.700, the PPI network comprised 482 nodes and 4215 interactive edges. Based on the topological analysis of the network, the average node degree was 17.5, and the average local clustering coefficient was 0.476. Data with an interaction score greater than 0.7 were imported into Cytoscape (version 3.7.1) to construct a PPI network (Figure 3).

To precisely identify the hub targets, the PPI network was analyzed by six important topological parameters: “BC”, “CC”, “DC”, “EC”, “NC”, and “LAC”. A node exhibiting high DC, BC, CC, EC, NC, and LAC values was deemed to have a significant impact within the network. Screening threshold was based on the median values of each parameter. The threshold values for the first screening were BC ≥ 215.410, CC ≥ 0.209, DC ≥ 12, EC ≥ 0.013, NC ≥ 5.682, and LAC ≥ 4.429. After the first screening, we obtained 129 hub nodes and 1899 edges. Subsequently, the threshold values of the second screening were BC ≥ 37.312, CC ≥ 0.542, DC ≥ 25, EC ≥ 0.064, NC ≥ 14.363, LAC ≥ 12.783. After that, 53 hub nodes and 792 edges were finally screened out (Figure 3).

#### 2.2.3. The Compound-Target Network

**Figure 4:**
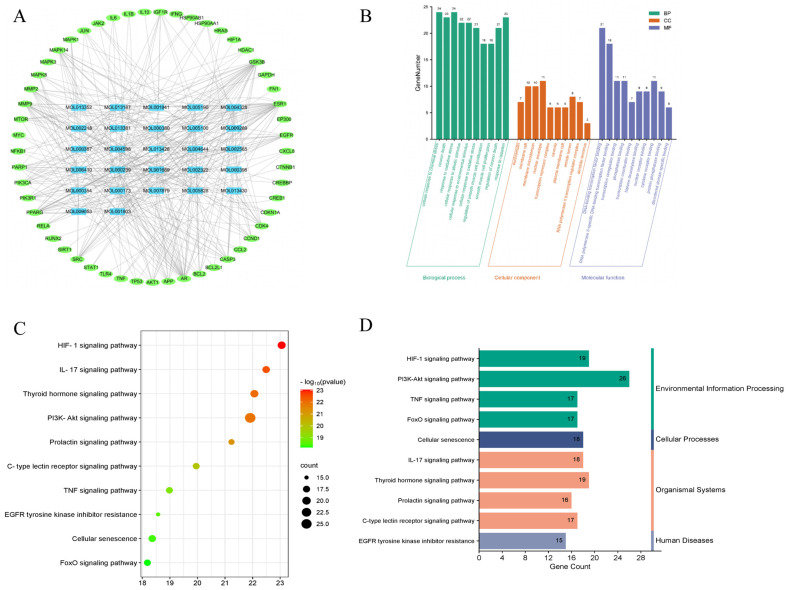
Compound-Target network construction and enrichment analyses. (**A**) The Compound-Target network. In the network, hub targets are denoted by green nodes, while active compounds are represented by blue nodes. The interactions between targets and compounds are shown by the edges. In this figure, Mol ID was used to represent each active compound (the corresponding relationships between Mol IDs and compounds are shown in Supplementary Table S7). (**B**) GO enrichment analysis of the hub targets. (**C**,**D**) KEGG enrichment analysis of the hub targets. (**C**) depicts the top 10 KEGG enrichment pathways. (**D**) depicts the KEGG pathway categorization.

A Compound-Target network with 27 compounds and 53 hub targets was constructed using Cytoscape 3.7.1 (Figure 4A), which can assist us in clearer understanding of the interactions between active compounds and the hub targets. The network contains a total of 80 nodes and 383 edges. CytoHubba, a plug-in for Cytoscape, was widely used to identify the important nodes in the network based on the parameters [23]. The important active compounds in the network, namely Wogonin (MOL000173), Nobiletin (MOL005828), Naringenin (MOL004328), Tetramethoxyluteolin (MOL007879), and Isorhamnetin (MOL000354), were identified using CytoHubba with the degree value serving as the screening criterion.

#### 2.2.4. Gene Ontology (GO) Analysis of Hub Targets

To elucidate the biological functions or molecular mechanisms of the 53 hub targets, GO enrichment analysis was carried out. The terms obtained from the GO enrichment analysis were categorized and annotated according to the three primary GO categories: molecular function (MF), cellular component (CC), and biological process (BP). Set *p* < 0.05 as the threshold, a total of 2322 GO items were obtained (Supplementary Table S5), of which 2141 were BP, 60 were CC and 121 were MF.

According to *p* value, 10 main biological processes related to MF, CC and BP were selected from small to large and presented in Figure 4. The findings showed that TFK mainly participated in cellular response to chemical stress (GO: 0062197), neuron death (GO: 0070997), response to oxidative stress (GO: 0006979), cellular response to abiotic stimulus (GO: 0071214), cellular response to environmental stimulus (GO: 0104004), cellular response to oxidative stress (GO: 0034599), regulation of smooth muscle cell proliferation (GO: 0048660), positive regulation of miRNA metabolic process (GO: 2000630), regulation of smooth muscle cell proliferation (GO: 0048660), smooth muscle cell proliferation (GO: 0048659), regulation of neuron death (GO: 1901214), response to radiation (GO: 0009314). Moreover, TFK affected central fatigue by regulating two principal MF terms, namely, DNA-binding transcription factor binding (GO: 0140297), RNA polymerase II-specific DNA-binding transcription factor binding (GO: 0061629). As for the CC, the potential target genes might reside in the euchromatin (GO:0000791), membrane raft (GO:0045121), membrane microdomain (GO: 0098857), etc. (Figure 4B).

#### 2.2.5. Kyoto Encyclopedia of Genes and Genomes (KEGG) Pathway Analysis of Hub Targets

KEGG enrichment analysis was conducted to determine the important pathways linked to 53 hub targets. Using a threshold of *p* < 0.05, TFK was identified as being involved in 175 KEGG pathways (Supplementary Table S6). The top ten significant KEGG pathways with the lowest *p* value were chosen by removing unrelated pathways such as “Kaposi sarcoma-associated herpesvirus infection” “Hepatitis B” and “Proteoglycans in cancer” (Figure 4C), which might be the core pathways of TFK against central fatigue. According to KEGG pathway classification (https://www.kegg.jp/kegg/pathway.html, accessed on 19 April 2024), the pathways were divided into four subgroups: human disorders, cellular processes, organismal systems, and environmental information processing (Figure 4D). Among them, the PI3K-AKT signaling pathway, which enriched the most genes, may play a greater role in the treatment of central fatigue.

### 2.3. TFK Ameliorates Abnormal Behavioral Symptoms in Rats with Central Fatigue

**Figure 5:**
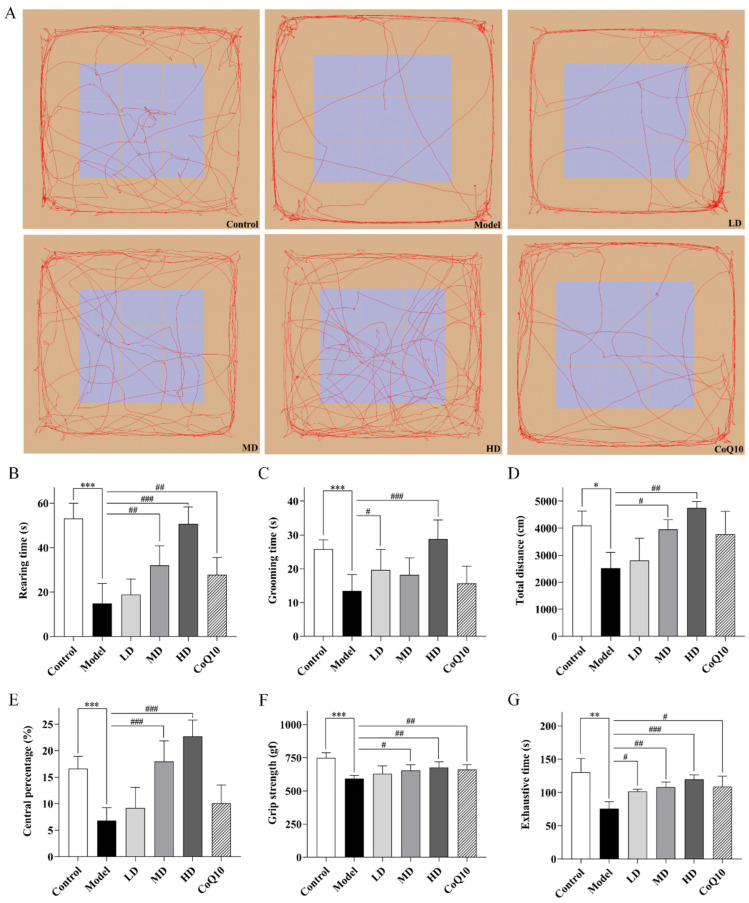
The locomotor activity decline and physical fatigue manifestations in central fatigue rats were evaluated through open field, grip strength, and forced exhaustive swimming tests to assess the efficacy of TFK in restoring their autonomous vitality. (**A**–**E**) Results of the open field test. Rearing time (**B**), grooming time (**C**), total distance (**D**), central percentage (**E**), and the representative movement trajectories of each group during a 5 min open field test session are presented (**A**). (**F**) Grip strength test was performed to detect skeletal muscle fatigue of rats. (**G**) Forced exhaustive swimming test was conducted to test the rats’ exercise endurance and evaluate their level of fatigue. Data are expressed as (mean ± SD), * *p* < 0.05, ** *p* < 0.01, *** *p* < 0.001 vs. control group (*n* = 6). ^#^ *p* < 0.05, ^##^ *p* < 0.01, ^###^ *p* < 0.001 vs. model group (*n* = 6).

Throughout the experimental period, no animal mortality or adverse events were observed. The results of the open field test revealed that in the central fatigue model rats, the time spent rearing, grooming, total distance traveled, and the percentage of distance traveled in the central zone all showed significant reductions in the open field box (*p* < 0.001, *p* < 0.001, *p* = 0.010, *p* < 0.001, Figure 5A–E). Both Coenzyme Q10 and high-dose of TFK could significantly extend the rearing time of central fatigue model rats (*p* = 0.008, *p* < 0.001). Meanwhile, high dose of TFK could also prolong the grooming time of the rats (*p* < 0.001). Furthermore, it was also observed that both medium and high doses of TFK increased the total motor distance and the percentage of the central region motor distance (*p* = 0.012, *p* = 0.001, *p* < 0.001, *p* < 0.001). The grip strength test and the forced exhaustive swimming test were employed to evaluate the degree of skeletal muscle fatigue and exercise endurance in rats. The results revealed a significant decrease in grip strength and time to exhaustion in rats with central fatigue (*p* < 0.001, *p* = 0.009, Figure 5F,G). Both medium and high doses of TFK and coenzyme Q10 were found to have enhancing effects (*p* = 0.020, *p* = 0.002, *p* = 0.009, *p* = 0.003, *p* < 0.001, *p* = 0.035), whereas the low-dose group only improved time to exhaustion (*p* = 0.018).

**Figure 6:**
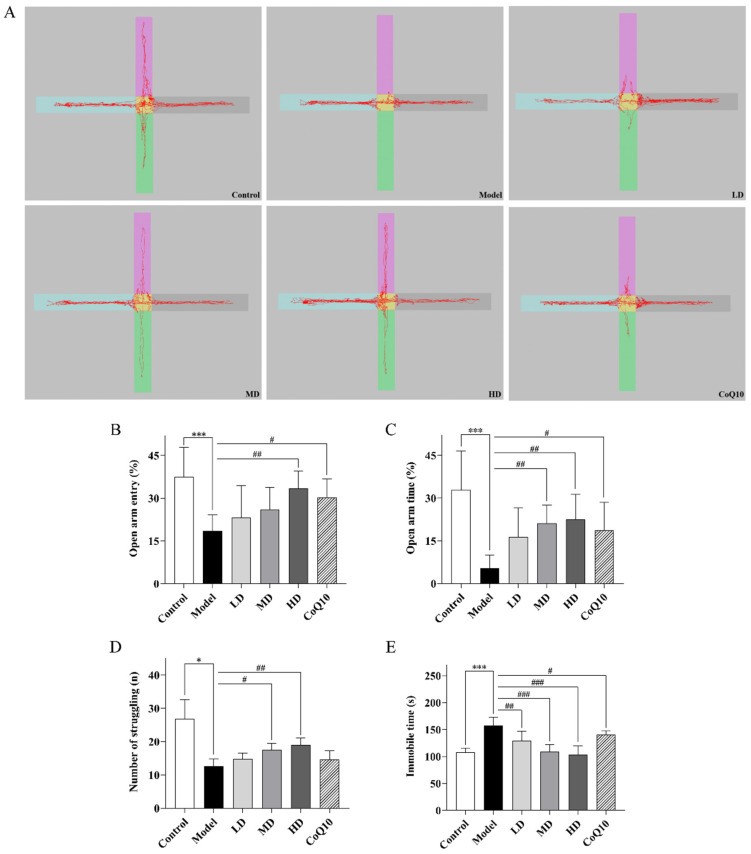
The assessment of emotional anomalies in central fatigue rats undergoing TFK was conducted through elevated plus maze test and tail suspension test. (**A**–**C**) Results of the elevated plus maze test. Open arm entry (**B**) and time (**C**) of rats on elevated plus maze, and representative movement trajectories for each group during the 5 min elevated plus maze test are illustrated (**A**). (**D**–**E**) Results of the tail suspension test. The number of struggling (**D**) and immobile time (**E**) of rats in tail suspension test are shown. Data were expressed as (mean ± SD), * *p* < 0.05, *** *p* < 0.001 vs. control group (*n* = 6). ^#^ *p* < 0.05, ^##^ *p* < 0.01, ^###^ *p* < 0.001 vs. model group (*n* = 6).

The results of the elevated plus maze test showed a significant decrease in the percentage of open arm entries and the percentage of time spent in the open arm for central fatigue model rats (*p* < 0.001, *p* < 0.001, Figure 6A–C). Both high-dose TFK and coenzyme Q10 could significantly increase the percentage of open arm entries and the percentage of time spent in the open arm for rats with central fatigue (*p* = 0.004, *p* = 0.018, *p* = 0.004, *p* = 0.022), indicating that both coenzyme Q10 and high-dose TFK effectively alleviated anxiety-like behavior in rats. Tail suspension test is regarded as one of the classic experiments for evaluating depressive-like behavior in rats [24]. Our results indicated a significant increase in immobility time for rats with central fatigue (*p* < 0.001, Figure 6D,E), suggesting the presence of depressive-like behavior. The low, medium, and high doses of TFK and Coenzyme Q10 could significantly reduce the immobility time of central fatigue rats (*p* = 0.001, *p* < 0.001, *p* < 0.001, *p* = 0.045). Moreover, medium and high doses of TFK also significantly increased the number of struggling attempts in the central fatigue rats (*p* = 0.035, *p* = 0.006).

**Figure 7:**
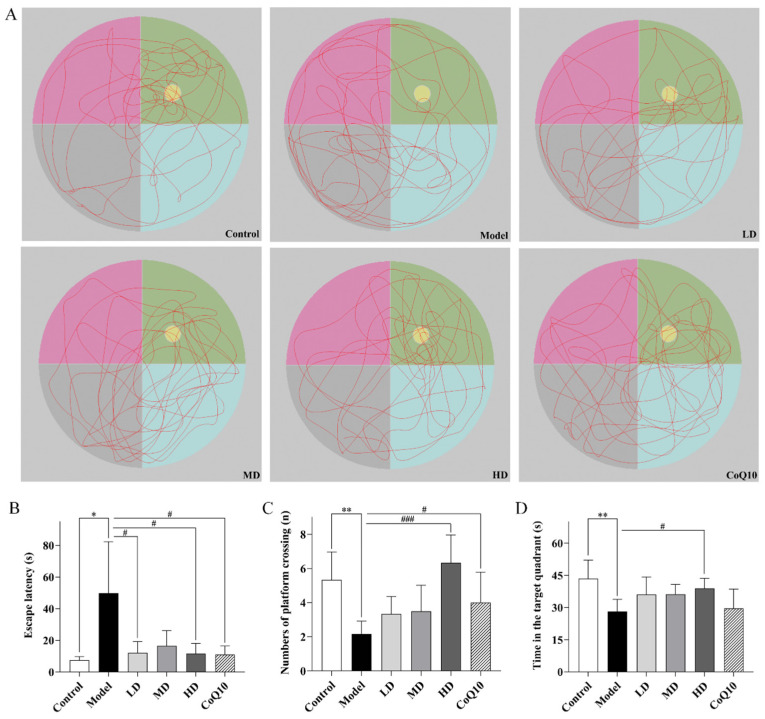
The evaluation of the cognitive functions improvement in central fatigue rats undergoing TFK was conducted through Morris water maze test. (**A**) Representative swim paths during the spatial probe test. The yellow circle indicates the location of the platform, while the green area denotes the first quadrant, which is the target quadrant. The escape latencies (**B**), numbers of platform crossings (**C**), the time in target quadrant (**D**) on the last day during Morris water maze test. Data were expressed as (mean ± SD), * *p* < 0.05, ** *p* < 0.01 vs. control group (*n* = 6). ^#^ *p* < 0.05, ^###^ *p* < 0.001 vs. model group (*n* = 6).

In the Morris water maze test, Figure 7A displayed the representative trajectories of rats during the memory probe phase within 2 min. The results indicated that rats in the central fatigue model exhibited prolonged escape latency, a significant decrease in the number of platform crossings, and reduced time spent swimming in the target quadrant (*p* = 0.025, *p* = 0.001, *p* = 0.001, Figure 7A–D), suggesting a significant decline in their learning and memory abilities. High doses of TFK and Coenzyme Q10 both significantly reduced the escape latency (*p* = 0.028, *p* = 0.029) and increased the number of platform crossings (*p* < 0.001, *p* = 0.035). Specifically, high doses of TFK also increased the time swimming in the target quadrant for rats with central fatigue (*p* = 0.018), demonstrating a restoration effect on learning and memory capabilities.

### 2.4. TFK Alleviates the Morphological Changes in the Hippocampal Tissues of Central Fatigue Rats

**Figure 8:**
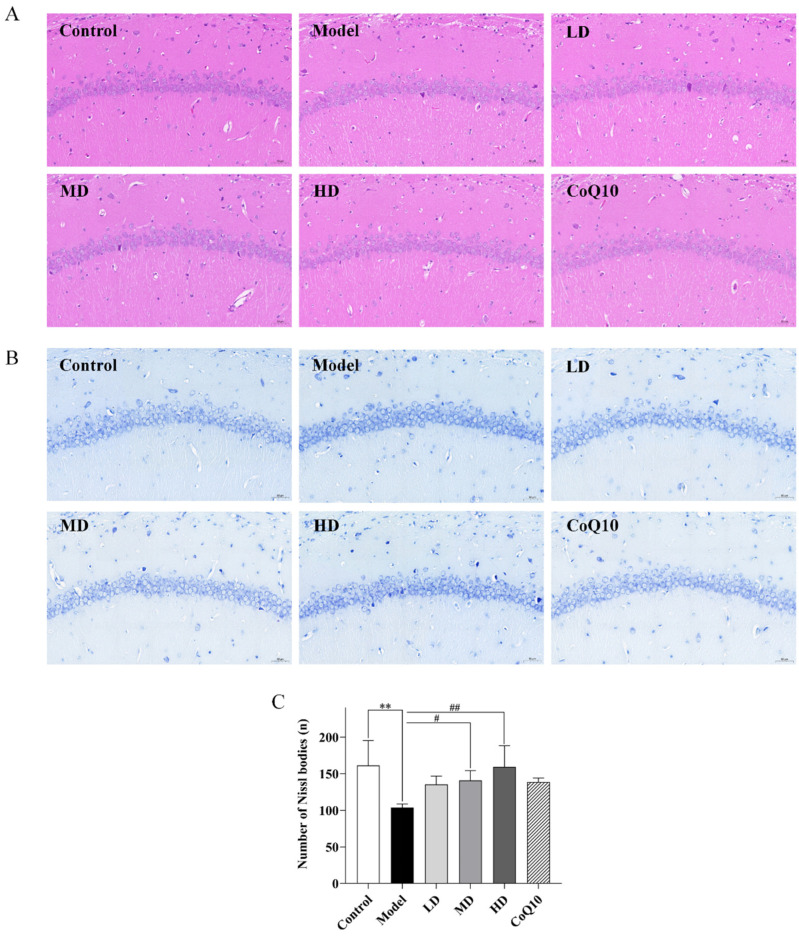
The assessment of morphological changes in the CA1 region of the hippocampal tissue. (**A**,**B**) HE staining and Nissl staining are shown (magnification ×200). (**C**) The number of Nissl bodies. The Nissl bodies were stained blue. Three fields were selected randomly in each sample for Nissl bodies calculation, and the numbers of Nissl bodies were averaged. Three samples were selected for each group. Data were expressed as (mean ± SD), ** *p* < 0.01 vs. control group (*n* = 3). ^#^ *p* < 0.05, ^##^ *p* < 0.01 vs. model group (*n* = 3).

Compared with the control group, rats in the central fatigue group showed partial neuron cell degeneration in the hippocampal tissues on HE staining, with deep staining indicating nuclear condensation and increased cellular basophilia. Low, medium, and high doses of TFK, along with coenzyme Q10, exhibited varying degrees of improvement in the pathological alterations in the hippocampal tissues of central fatigue rats. Notably, the high-dose group demonstrated the most significant therapeutic efficacy, as evidenced by tightly regulated neuronal cell arrangement in the hippocampal CA1 region and a notable decrease in neuron cell degeneration. Through Nissl staining, a significant decrease in Nissl bodies was observed in the hippocampus of central fatigue rats (*p* = 0.004, Figure 8A,B), suggesting potential neuron death and impairment of normal function. Meanwhile, both medium and high doses of TFK significantly increased the number of Nissl bodies in the hippocampal tissues of rats with central fatigue (*p* = 0.041, *p* = 0.005, Figure 8C), indicating their potential neuroprotective effects on neurons.

### 2.5. TFK Ameliorates Oxidative Stress Damage in the Hippocampal Tissues Involving PI3K-AKT Signaling Pathway

**Figure 9:**
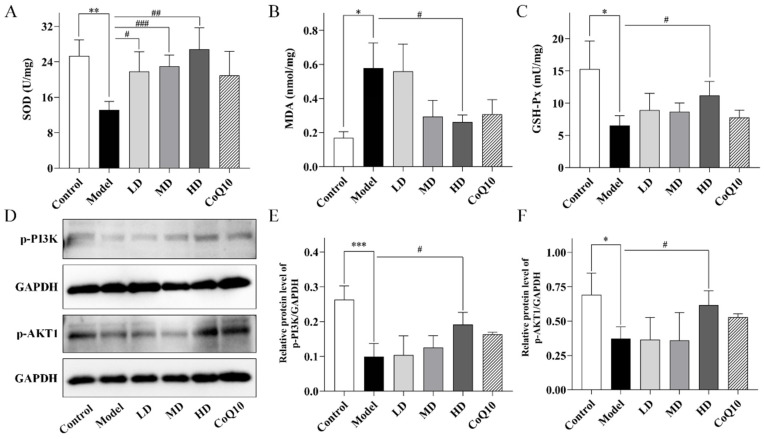
TFK ameliorates oxidative stress damage in the hippocampal tissues via PI3K-AKT signaling pathway. (**A**–**C**) The expression levels of SOD, MDA, GSH-Px. (**D**) Representative Western Blot bands for target proteins phospho-PI3K, phospho-AKT1 and internal reference protein GAPDH. (**E**,**F**) Quantification results for the ratio of phospho-PI3K and phospho-AKT to GAPDH, respectively. Data were expressed as (mean ± SD), * *p* < 0.05, ** *p* < 0.01, *** *p* < 0.001 vs. control group ((**A**–**C**): *n* = 6, (**E**,**F**): *n* = 3). ^#^ *p* < 0.05, ^##^ *p* < 0.01, ^###^ *p* < 0.001 vs. model group ((**A**–**C**): *n* = 6, (**E**,**F**): *n* = 3).

Immediately following, the oxidative stress process and PI3K-AKT signaling pathway, which were predicted by network pharmacology to play key roles in TFK’s intervention against central fatigue, were experimentally validated. Compared with the control group, a significant increase in malondialdehyde (MDA) was observed in the hippocampal tissue of central fatigue rats (*p* = 0.011, Figure 9B), while the activities of superoxide Dismutase (SOD) and glutathione peroxidase (GSH-Px) were markedly reduced (*p* = 0.001, *p* = 0.048, Figure 9A,C), which suggested the occurrence of oxidative stress damage in the brains of central fatigue rats. The low, medium, and high doses of TFK all significantly increased the SOD activity in the hippocampal tissues of central fatigue rats (*p* = 0.048, *p* < 0.001, *p* = 0.007). The high dose of TFK also significantly enhanced the GSH-Px activity (*p* = 0.030) and reduced the MDA levels (*p* = 0.037), thereby enhancing antioxidant capacity and reducing the risk of hippocampal neuron damage. Furthermore, significantly decreased phosphorylation levels of PI3K and AKT1 were observed in the hippocampal tissues of rats with central fatigue (*p* < 0.001, *p* = 0.014, Figure 9D–F), suggesting that central fatigue impairs PI3K/AKT1 signaling activity. Notably, high-dose TFK treatment markedly restored the phosphorylation levels of both PI3K and AKT1 (*p* = 0.011, *p* = 0.049, Figure 9D–F). These results demonstrated that TFK may attenuate oxidative stress damage in the hippocampus of centrally fatigued rats by activating the PI3K-AKT signaling pathway.

### 2.6. TFK Modulates Neurotransmitter Levels and Biochemical Metabolic Markers in Central Fatigue Rats

**Figure 10:**
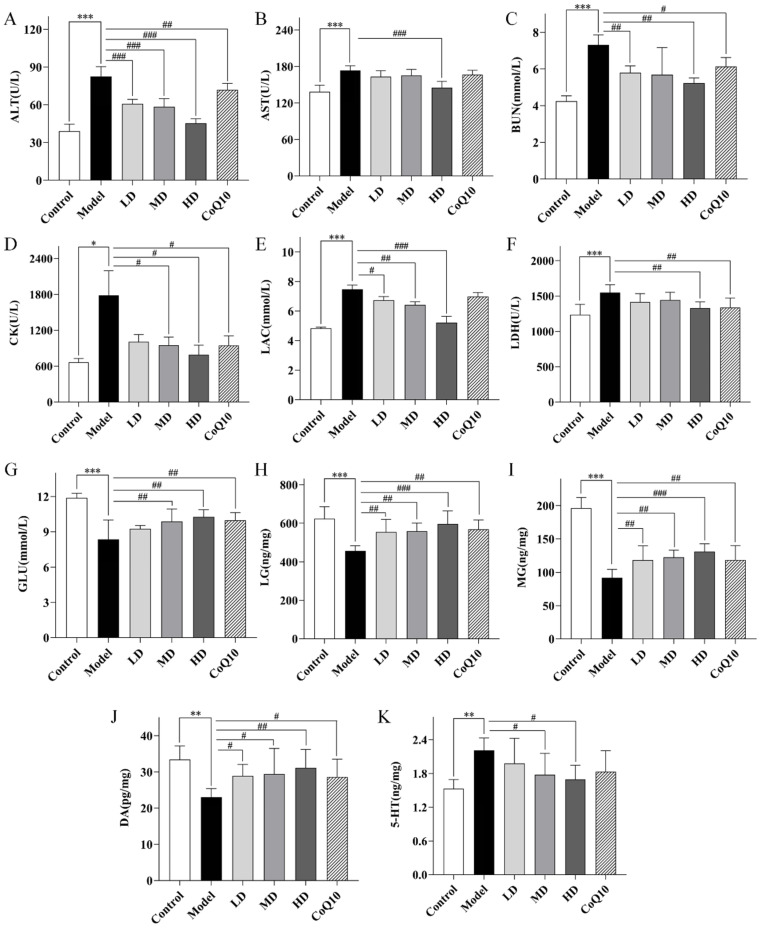
The regulation of metabolic function and neurotransmitter secretion in the hippocampal tissue of central fatigue rats undergoing TFK was explored. (**A**–**G**) Serum concentrations of ALT, AST, BUN, CK, LAC, LDH and GLU were quantified with an automatic biochemical analyzer. (**H**–**K**) The expression levels of LG, MG, DA and 5-HT were tested by ELISA kits. Data were expressed as (mean ± SD), * *p* < 0.05, ** *p* < 0.01, *** *p* < 0.01 vs. control group (*n* = 6). ^#^ *p* < 0.05, ^##^ *p* < 0.01, ^###^ *p* < 0.001 vs. model group (*n* = 6).

The concentrations of monoamine neurotransmitters,5-hydroxytryptamine (5-HT), and dopamine (DA), in the hippocampal tissues were quantified using the Enzyme-Linked Immunosorbent Assay (ELISA) method. Compared with the control group, the level of 5-HT increased (*p* = 0.001, Figure 10K) and the level of DA decreased (*p* = 0.001, Figure 10J) in the hippocampal tissues of central fatigue rats, indicating the presence of abnormal neurotransmitter secretion. Both medium and high doses of TFK were effective in reducing the levels of 5-HT (*p* = 0.027, *p* = 0.010) and increasing the levels of DA (*p* = 0.023, *p* = 0.005).

Moreover, compared with the control group, the rats in the central fatigue group showed significant increase in the peripheral blood levels of alanine aminotransferase (ALT), aspartate aminotransferase (AST), blood urea nitrogen (BUN), creatine kinase (CK), lactate (LAC), and lactate dehydrogenase (LDH) (*p* < 0.001, *p* < 0.001, *p* < 0.001, *p* = 0.015, *p* < 0.001, *p* < 0.001, Figure 10A–F), indicating abnormalities in multiple metabolic pathways such as amino acid and glycolysis. High-dose of TFK exhibited significant reversal effects on the aforementioned abnormal indicators (*p* < 0.001, *p* < 0.001, *p* = 0.001, *p* = 0.018, *p* < 0.001, *p* = 0.003), indicating its potential to ameliorate metabolic disorders in rats with central fatigue. In contrast, medium and low doses of TFK and Coenzyme Q10 could only improve part of the abnormal indicators, indicating that high-dose TFK had the most effective regulatory effect on metabolic disorders. Therefore, the high-dose group was chosen for subsequent metabolomics study. A decrease in glucose (GLU), liver glycogen (LG), and muscle glycogen (MG) was also observed in rats with central fatigue (*p* < 0.001, *p* < 0.001, *p* < 0.001, Figure 10G–I). Both the medium dose and high dose groups were able to increase blood glucose levels (*p* = 0.006, *p* = 0.001) and restore hepatic glycogen and muscle glycogen reserves (*p* = 0.003, *p* < 0.001, *p* = 0.003, *p* < 0.001).

### 2.7. Untargeted Serum Metabolomics Analysis

**Figure 11:**
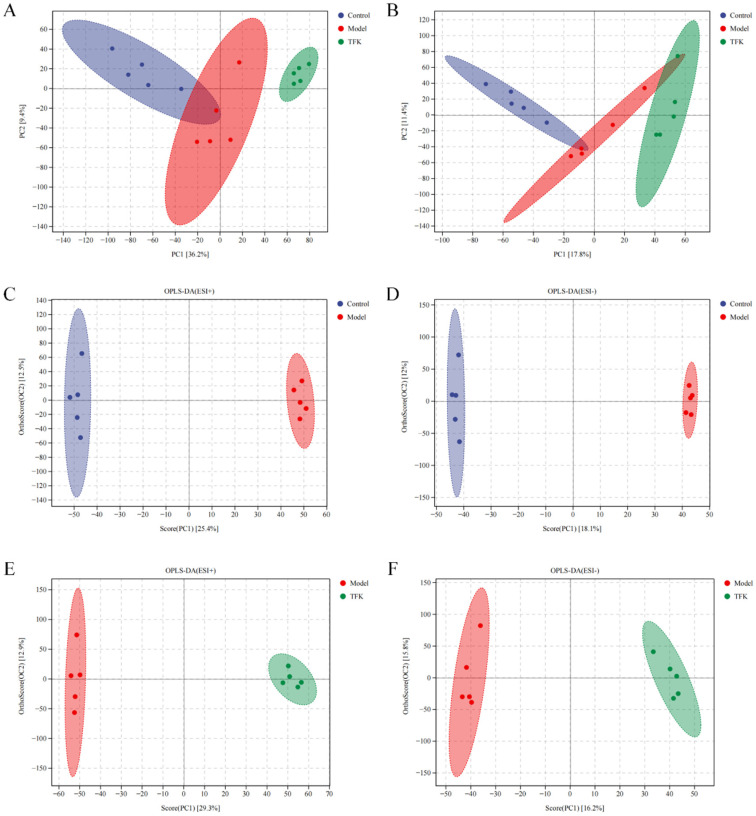
Principal component analysis (PCA) and multivariate statistical PLS-DA (OPLS-DA) analysis were conducted on three groups of samples in both ESI- and ESI+ modes. (**A**) PCA in ESI+ mode. (**B**) PCA in ESI- mode. (**C**,**D**): OPLS-DA analysis of control and model groups in both ESI- and ESI+ modes. (**E**,**F**) OPLS-DA analysis of model and TFK groups in both ESI- and ESI+ modes.

Untargeted serum metabolomics analysis was conducted on rats from the blank control group, model group, and TFK group (the high dose was chosen). Principal component analysis (PCA) was employed to discern clustering trends among the blank control group, model group, and TFK group (Figure 11A,B). Orthogonal-partial least squares discriminant analysis (OPLS-DA) was conducted to explore the underlying structures between groups (Figure 11C–F). In positive ion mode, the R and Q coefficients between the control and model groups were 0.999 and 0.786, respectively, while between the model and TFK groups they were 0.998 and 0.860. In negative ion mode, the R and Q coefficients between control and model groups were 1.000 and 0.673, respectively, whereas between the model and TFK groups they were 0.994 and 0.503. These results indicated that there were significant differences in metabolite levels among the control, model and TFK groups.

**Figure 12:**
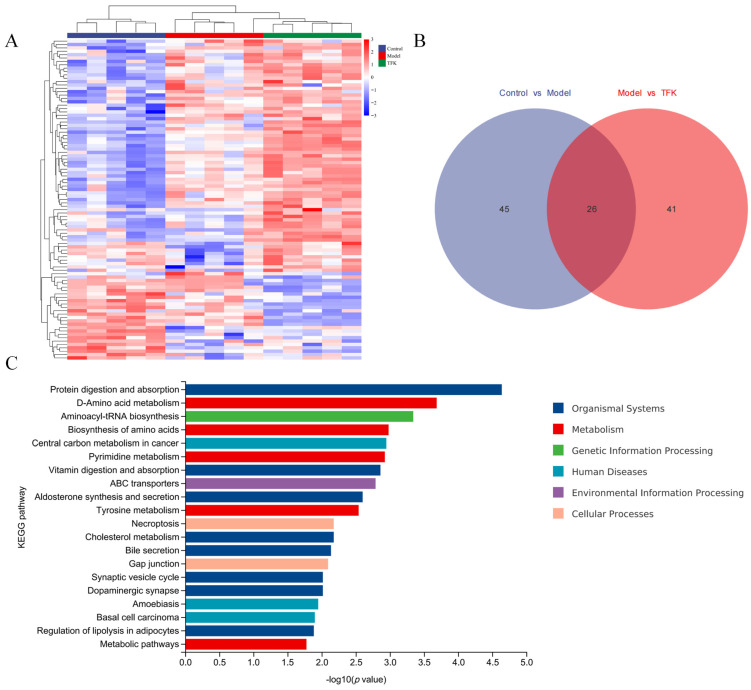
Serum metabolomics differential metabolite analysis. (**A**) Differential substance clustering analysis. (**B**) Venn analysis of differential metabolites among three groups. (**C**) KEGG enrichment analysis of differential metabolites.

Metabolites with a variable importance in projection score greater than 1 and a statistically significant *t*-test result with *p* < 0.05 were identified as potential biomarkers. After identifying all biomarkers through the Human Metabolome database, a total of 303 metabolites were identified. Of them, a total of 71 metabolites were significantly different between the control group and the model group, while 67 metabolites were significantly different between the model group and the TFK group, and 26 metabolites exhibited significant differences among the control group, the model group, and the TFK group (Figure 12A,B). By conducting KEGG enrichment analysis on the 26 differential metabolites, relevant metabolic pathways such as Protein digestion and absorption, D-amino acid metabolism, Aminoacyl-tRNA biosynthesis, Biosynthesis of amino acids, Tyrosine metabolism, synaptic vesicle cycle, and dopaminergic synapse were identified (Figure 12C).

**Figure 13:**
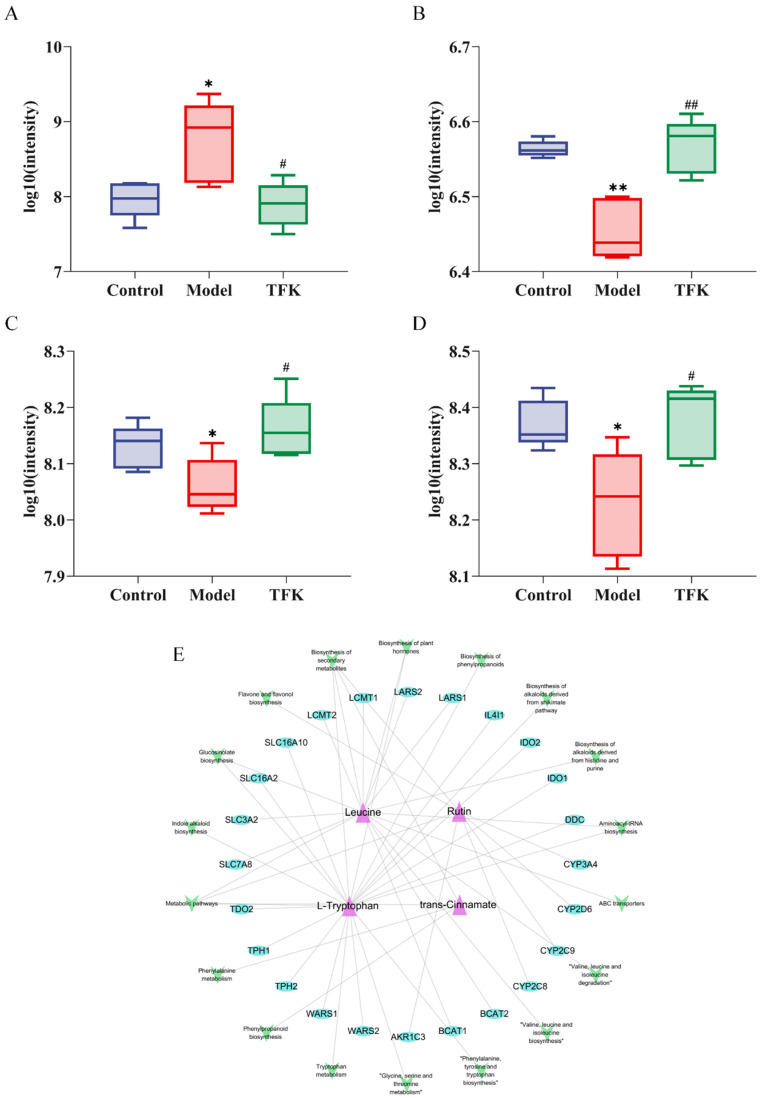
Analysis of the four reverse differential metabolites. (**A**–**D**) Analysis of the changing trends of reverse metabolites among three groups: rutin (**A**), trans-Cinnamate (**B**), Leucine (**C**), and L-Tryptophan (**D**). (**E**) Metabolic biomarker-target-pathway network of the four reverse metabolites. * *p* < 0.05, ** *p* < 0.01; *^#^ p* < 0.05, *^##^ p* < 0.01.

Moreover, TFK treatment led to a reversal of the significant alterations observed in four metabolites between the model and control groups. These metabolites were rutin, leucine, trans-cinnamate, and L-tryptophan, as shown in Figure 13. These four metabolites have the potential to act as important metabolic biomarkers for TFK in treating central fatigue. Through MBROLE platform, a total of 18 metabolic pathways and 24 proteins were found to be associated with the four metabolites. Specific metabolic pathways and protein information can be found in the biomarker-target-pathway network (Figure 13). In this network, leucine and L-tryptophan possess a high number of connections and can therefore be regarded as core metabolic biomarkers.

## 3. Discussion

With the evolution of lifestyle and life rhythm, central fatigue is increasingly emerging as a significant factor impacting individuals’ lives, work, and studies in comparison to peripheral fatigue primarily induced by excessive work [25]. TCM offers the advantages of high safety and good efficacy in treating central fatigue [16]. In this study, the considerable therapeutic potential of TFK decoction in alleviating central fatigue was demonstrated. The integration of network pharmacology and metabolomics has gradually been established as a research paradigm in the field of TCM [26]. Using this combined approach, the underlying mechanisms of TFK decoction were explored, and it was revealed that central fatigue may be mitigated through the targeting of oxidative stress, neuronal death, and the PI3K-AKT signaling pathway. Furthermore, it was suggested that the regulation of amino acid metabolism constitutes a core metabolic mechanism responsible for the alleviation of central fatigue-related symptoms by TFK.

Central fatigue arises from prolonged mental stress and other factors that result in central nervous system dysfunction, leading to declined cognitive function, reduced memory capacity, decreased physical endurance, increased negative emotions such as anxiety and depression, as well as various metabolic disturbances in both central and peripheral systems [27]. The assessment of rats through a series of behavioral tests is crucial in central fatigue research and its reliability is well established [28,29,30]. The results from the grip strength test and forced exhaustive swimming test indicated a decrease in exercise endurance among the central fatigue model rats. Meanwhile, the results from open field experiments and elevated maze tests demonstrated a significant decrease in exploratory behavior and an accompanying high level of anxiety-related behaviors in the model group of rats. The tail suspension test results suggest that the model group of rats exhibited clear signs of depression. t In the Morris water maze test, the model rats showed a decrease in the number of crossings over the platform, time spent in the target quadrant, and distance swum in the target quadrant, indicating their inability to accurately remember the location of platform. In contrast, TFK treatment produced significant improvements in all tested behavioral parameters. These improvements manifested as heightened exploratory behavior, reduced negative emotions, and enhanced physical and memory capabilities, suggesting that TFK can effectively mitigate the behavioral deficits associated with central fatigue.

To further explore the potential mechanisms of TFK in treating central fatigue, the methods of network pharmacology and metabolomics were utilized. First, a total of 210 compounds were identified in TFK using UHPLC-MS/MS method. Based on the compound identification results, 30 active compounds and 685 potential targets of TFK were screened out. After intersecting with 5529 central fatigue-related targets, a total of 482 common targets of TFK and central fatigue were identified. Subsequent PPI network construction and analysis refined the 482 common targets to 53 core targets. GO enrichment analysis revealed their primary impact on biological processes such as neuron death and oxidative stress. Brain is the central regulator of fatigue [31]. A close association has been found between neuronal function and central fatigue, with studies indicating that central fatigue can significantly increase the risk of neuron death [11]. The research findings also demonstrated the presence of significant brain neuron damage and death in central fatigue rats. TFK can significantly alleviate brain neuron damage and death in central fatigue rats. The augmentation of cerebral oxidative stress is a key inducer of mental fatigue [32]. Previous study has indicated significant oxidative stress damage in the brain of central fatigue rats, which parallels with findings of this current study [30]. Meanwhile, it was revealed that the activities of SOD and GSH-Px in the brains of central fatigue rats were significantly enhanced by a high dose of TFK, while MDA levels were decreased, thereby alleviating oxidative stress damage in the brain. CK levels serve as a crucial indicator for assessing fatigue, as its activation in the oxidative stress process can scavenge oxygen free radicals, thereby mitigating oxidative stress damage that contributes to central fatigue [33,34]. It was revealed that TFK can significantly elevate the levels of CK in the peripheral blood of rats subjected to a central fatigue model. KEGG enrichment analysis indicated that the PI3K-AKT signaling pathway may serve as the key pathway for TFK in treating central fatigue. The experimental results verified that TFK significantly activates the core targets PI3K and AKT1 in the PI3K-AKT signaling pathway through phosphorylation. Substantial evidence demonstrates that activation of the PI3K-AKT signaling pathway is closely associated with alleviating oxidative stress damage. For example, in diabetic encephalopathy, activation of the PI3K/AKT pathway can reduce oxidative stress-induced brain injury [35]. Similarly, in human cardiomyocytes, activation of the PI3K/AKT pathway has been shown to mitigate oxidative stress damage caused by ischemia–reperfusion injury [36]. Therefore, it was proposed that the pharmacological effects of TFK are achieved via activation of the PI3K/AKT signaling pathway, thereby reducing hippocampal oxidative stress damage induced by central fatigue and ultimately resulting in neuroprotection.

Currently, it is believed that modulating amino acid metabolism, lipid metabolism, and glucose metabolism disorders may serve as the potential pathways to improve fatigue symptoms [37]. LAC serves as a crucial intermediate product of glucose metabolism, while urea nitrogen is an important metabolic product of amino acids [38,39]. AST and ALT play crucial roles in the amino acid metabolism and serve as significant markers of liver function [40,41]. Numerous studies have indicated that fatigue can induce substantial changes in the levels of metabolic substances such as LAC, BUN, ALT and AST in the peripheral blood [39,42,43]. This study revealed a significant decrease in peripheral blood glucose level and a notable increase in the level of BUN, LAC, ALT and AST in central fatigue model rats. Additionally, the level of LDH was markedly reduced. Meanwhile, a high dosage of TFK was able to significantly reverse these changes in central fatigue model rats, which indicated that TFK can play a significant regulatory role in the metabolic disturbances and liver function of the central fatigue model rats.

To further explore the global metabolic alterations in central fatigue model rats and explore the possible metabolic-based mechanism of TFK, a metabolomics study was performed. Significant differences were observed in 71 serum metabolites between the control and model groups, in 67 serum metabolites between the model and TFK groups, and in 26 metabolites across the control, model, and TFK groups. These 26 metabolites were primarily enriched in pathways related to amino acid metabolism, such as D-amino acid metabolism, biosynthesis of amino acids and tyrosine metabolism. Furthermore, the results also revealed significant enrichment of mechanisms related to synaptic vesicle cycle and dopaminergic synapse, which are associated with neuron synapses. Studies have indicated that significant changes in synapses are closely associated with the occurrence of fatigue symptoms [44,45]. Moreover, the presynaptic neuronal AChRs play important roles in the release of neurotransmitters such as DA and 5-HT [46]. Currently, one hypothesis regarding the central fatigue etiology is that alterations in energy and amino acid metabolism induced by exercise indirectly lead to increased brain tryptophan uptake and 5-HT synthesis and release, causing an imbalance between 5-HT and DA, consequently resulting in the manifestation of central fatigue related symptoms, such as depression and anxiety [47,48,49]. In this study, a significant increase in 5-HT levels and a significant decrease in DA levels were observed in the central fatigue model group of rats exhibiting anxiety-like behaviors. The intervention of high-dose TFK effectively reversed these alterations, which demonstrated that TFK can significantly ameliorate the imbalance between brain 5-HT and dopamine caused by abnormal amino acid metabolism.

Further analysis of the metabolomic data revealed that rutin, leucine, trans-cinnamate, and L-tryptophan were significantly reversed metabolite components after intervention with TFK. These four metabolites may serve as the important potential metabolic biomarkers for central fatigue during TFK intervention. After constructing a metabolic biomarker-target-pathway network, it was revealed that L-tryptophan and leucine may serve as the core important metabolic biomarkers within this network. Previous study has confirmed that leucine can alleviate central fatigue by promoting muscle protein synthesis [50]. In this study, a significant decrease in the level of leucine was observed in the peripheral blood of central fatigue rats. Nevertheless, the usage of TFK was able to significantly reverse this trend, indicating that TFK may exert anti-fatigue effects through elevating leucine levels. L-tryptophan is a precursor to the synthesis of 5-HT and the synthesis of 5-HT may consume L-tryptophan [51]. The study results indicated a significant decrease in peripheral blood levels of L-tryptophan in central fatigue rats, suggesting a significant enhancement in brain 5-HT synthesis. After the TFK intervention, a significant increase was observed in the level of L-tryptophan compared to the model group, indicating a potential inhibition on brain 5-HT synthesis process, which is beneficial for correcting the imbalance between 5-HT and DA in the brain caused by central fatigue.

This study has several key innovations. For the first time, it was proposed and preliminarily validated through an approach integrating network pharmacology with experimental verification that the “PI3K-AKT pathway-mediated alleviation of hippocampal oxidative stress” is one of the potential mechanisms by which TFK treats central fatigue. Furthermore, this research represents the first serum metabolomics analysis conducted on a central fatigue rat model under TFK intervention. It identified potentially regulated metabolic pathways and biomarkers, and by constructing a metabolic biomarker-target-pathway network, revealed two core important biomarkers: L-tryptophan and leucine. Integrated with measurements of 5-HT and DA, an innovative potential pathway is proposed: TFK may modulate peripheral L-tryptophan levels, thereby indirectly influencing its conversion to 5-HT in the brain, while coordinately regulating leucine metabolism, collectively acting to restore the balance of 5-HT and DA in the hippocampus.

This study still has some shortcomings that must be further addressed. First, the core targets within the pathways predicted by network pharmacology have not been subjected to further in-depth experimental validation using techniques such as gene overexpression or knockout. Second, the chemical components in the TFK sample were only identified through the UHPLC-MS/MS method, without directly measuring the actual brain-penetrating components. Thus, it was unable to definitively ascertain the specific components through which TFK exerts its effects on the brain. Finally, this study only employed metabolomics as the single omics research approach, which may be considered somewhat limited in the exploration of underlying mechanisms. Future investigations will employ integrated transcriptomic and proteomic analyses to more comprehensively explore the mechanisms underlying TFK therapy for central fatigue. Moreover, the crucial signaling pathways and molecular targets identified in this study require deeper experimental validation to definitively elucidate how TFK exerts its core therapeutic effects against central fatigue. Additionally, the determination of the brain-entry chemical constituents of TFK will be necessary to ascertain the pharmacological components responsible for its effects on cerebral cells.

## 4. Materials and Methods

### 4.1. TFK Preparation and UHPLC-MS/MS Analysis

The herb components of TFK were purchased from Tongrentang (Tongrentang Pharmaceutical Co., Ltd., Beijing, China), specifically including 18 g of Huang Qi (*Astragali Radix*), 12 g of Zhi Qiao (*Aurantii Fructus*), 12 g of Shan Zha (*Crataegi Fructus*), 18 g of Chai Hu (*Bupleuri Radix*), and 6 g of Dang Gui (*Angelicae Sinensis Radix*). The botanical identification of each herb in TFK decoction was conducted by Professor Tian Guofang and Professor Kang Yu from the Pharmaceutical Technology Center at the School of Pharmaceutical Sciences, Tsinghua University. The proportion of herbs in TFK were selected based on the prescription approved by the Chinese Scientific and Technological Achievements Registration System (Registration Number: 39430140). After weighing, the five herbs were soaked in water for 30 min, boiled over high heat (heating with 600 W power), then simmered at a low heat (heating with 300 W power) to maintain a slight boil for 1.5 h. Afterward, the mixture was filtered through gauze to obtain the filtrate. This process was repeated twice, combining the filtrates each time. The combined filtrates were then concentrated to a solution with a concentration of 0.2 g/mL to prepare the TFK decoction solution, which was stored in a medical-grade ultra-low temperature refrigerator [52]. The TFK sample (200 mg) was placed in a 15 mL centrifuge tube and extracted with 10 mL of a 50:50 (*v*/*v*) methanol/water solution.

After ultrasonicated for 30 min, 1 mL of the supernatant was collected and centrifuged at 14,000 rpm for 5 min. After filtering through a 0.22 μm filter membrane, the supernatant was transferred to sample vial for UHPLC-MS/MS analysis. Chromatographic separation was achieved using an ACQUITY UPLC HSS T3 column with the dimensions of 2.1 mm × 100 mm and a particle size of 1.8 μm. The mobile phases consisted of (A) deionized water with 0.1% formic acid and (B) acetonitrile with 0.1% formic acid. A gradient elution program was applied under the following conditions: 0 min, 100% A and 0% B; 15 min, 80% A and 20% B; 50 min, 0% A and 100% B; 60 min, 0% A and 100% B; 70 min, 100% A and 0% B. The column temperature was maintained at 35 °C and the flow rate was set at 0.25 mL/min.

### 4.2. Network Pharmacology Analysis

#### 4.2.1. Active Compounds and Targets of TFK

The compounds of TFK were obtained through UHPLC-MS/MS analysis. ADME (absorption, distribution, metabolism, excretion) is an important drug evaluation method which is commonly applied for finding the active compounds of drugs. In this study, active compounds were selected based on the ADME criteria of OB ≥ 30% and DL ≥ 0.18. OB denotes the fraction of an orally administered drug that reaches the systemic circulation, serving as a critical parameter in the pharmacokinetic ADME profile [53]. DL is an established concept for predicting the drug-like property of the compounds [54]. The targets of the active compounds were obtained from five databases: Traditional Chinese Medicine Systems Pharmacology Database and Analysis Platform (TCMSP, https://tcmsp-e.com/tcmsp.php, accessed on 17 April 2024), Herb (http://herb.ac.cn/, accessed on 17 April 2024), SwissTargetPrediction (URL https://www.swisstargetprediction.ch/, accessed on 17 April 2024), DrugBank (URL https://www.drugbank.com/, accessed on 17 April 2024), and STITCH (http://stitch.embl.de/, accessed on 17 April 2024). The TCMSP platform serves as a crucial tool for examining the connections among Chinese herbal medicines, their corresponding targets, and the disorders they address, employing a systems pharmacology approach that encompasses chemicals, targets, drug-target networks, and drug-target-disease networks [55]. Herb is a natural medicine database platform that aggregates both high-throughput experimental data and information extracted from scientific references. It provides functionalities for browsing, searching, viewing, and downloading data related to TCM herbs, their active ingredients, target genes, diseases, high-throughput experiments, and mined literature [56]. SwissTargetPrediction allows for researchers to estimate the most probable macromolecular targets of a small molecule, assumed as bioactive [57]. DrugBank is a public online database containing biochemical information about drugs and their gene targets [58]. STITCH serves as an interaction network database for small molecules and proteins [59]. The UniProt database was used to normalize the targets related to the active compounds [60].

#### 4.2.2. Disease Targets

“Central fatigue,” “Central nervous system fatigue,” and “Center fatigue,” were used as the search keywords to retrieve disease targets associated with central fatigue from The Human Gene Database (GeneCards) [61], The Online Mendelian Inheritance in Man (OMIM) [62], and The Pharmacogenomics Knowledgebase (PharmGKB) databases [63]. In the GeneCards database, targets with a Relevance score greater than or equal to the median were defined as potential central fatigue-related targets. The targets related to central fatigue were finally achieved by combining the results of the three databases and removing the repeated targets.

#### 4.2.3. Common Targets Screening

Jvenn is an interactive Venn diagram viewer designed for efficient comparison of diverse result sets and extraction of common elements. In our study, Jvenn was employed to generate a Venn diagram and identify common targets shared between TFK and central fatigue. These common targets were considered as the potential therapeutic targets for TFK against central fatigue.

#### 4.2.4. PPI Network

PPI network can help us gain a deeper understanding about how proteins interact in complex diseases such as central fatigue. The string database (https://string-db.org/, accessed on 19 April 2024) [64] was used to find the protein–protein interaction between potential therapeutic targets for TKF when the species was set to *Homo sapiens*. Cytoscape (version 3.7.1) was widely utilized in network pharmacology research as a robust open-source platform for the analysis and visualization of biological networks [23]. In this study, Cytoscape was used to establish and visualize the protein–protein interaction network. The hub targets in the PPI network were obtained by topological screening in Cytoscape.

#### 4.2.5. Compound-Target Network

The compound-target network was established to elucidate the functional mechanism underlying TFK. Effective compounds of TFK and hub targets identified from the PPI network were first organized in Excel and subsequently imported into Cytoscape to construct a compound–target network for visualization.

#### 4.2.6. GO and KEGG Enrichment Analyses

The clusterProfiler package [65] and R (version 4.0.3) were used to perform GO enrichment and KEGG enrichment analyses. After filtering out irrelevant pathways, the most significantly enriched GO terms and KEGG pathways (using *p* < 0.05 as the threshold) were selected based on lowest *p*-value. GO enrichment analyses are typically used to identify predominant processes, while KEGG pathway enrichment analyses represent the gene-related pathways and molecular interactions.

### 4.3. Animal Grouping and Model Establishment

**Figure 14:**
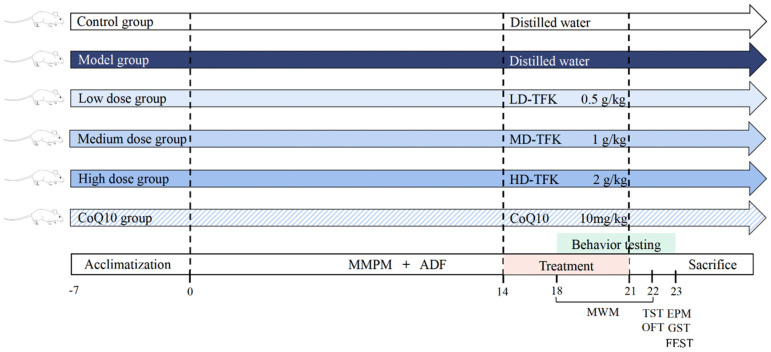
Experimental procedure. In this study, after a 7-day acclimation period, all rats except the blank control group were subjected to a 21-day central fatigue model establishment using the MMPM combined with ADF method. From days 14 to 21, the blank control and model groups received distilled water, while the treatment groups were administered their respective drugs. Behavioral assessments were conducted over days 18–23, which included the MWM from days 18 to 21. The TST and OFT were performed on day 22, followed by the EPM, GST, and FEST on day 23. All animals were sacrificed one day after the final behavioral test. MMPM, modified multiple platform method; ADF, alternate-day fasting; MWM, Morris Water Maze; TST, Tail Suspension Test; OFT, Open Field Test; EPM, Elevated Plus Maze; GST, Grip Strength Test; FEST, Forced Exhaustive Swimming Test.

Ninety, 8-week-old, male, specific-pathogen-free (SPF) Wistar rats weighing 200 ± 20 g were obtained from the Beijing SPF Biotechnology Co., Ltd. (Beijing, China, license number: SCXK 2019-0010). As an exploratory investigation, the optimal sample size determination was performed using the resource equation method while also meeting the minimum sample size requirements for the statistical analysis of all outcome measures [66]. The animal experiment procedures were approved by the Animal Experimentation Ethics Committee of Beijing University of Chinese Medicine (No. BUCM-2023090410-3777). Experimental animals were maintained under SPF conditions at the Animal Experimental Center of Beijing University of Chinese Medicine. Before the experiments, all rats were acclimated for 7 days under standard and identical housing conditions with a temperature of 23 ± 2 °C, relative humidity of 35 ± 5%, and a 12 h light/dark cycle, while having free access to water ad libitum. After the adaptive rearing period, rats that showed no signs of pre-existing illness or injury were included in the study and randomly allocated into six groups using a computer-based random number generator to ensure an unbiased distribution, with 15 rats in each group. Rats in control and model groups received daily administration of distilled water. Rats in coenzyme Q10 (CoQ10) group were treated with coenzyme Q10 (Eisai China Pharmaceutical Co., Ltd., Suzhou, China) by gavage (10 mg/kg/day). Coenzyme Q10 is a commonly used medication for fatigue treatment [67]. Based on the clinically recommended daily therapeutic dose recorded in the Chinese Scientific and Technological Achievements Registration System (Number: 39430140), the equivalent daily dose of TFK for rats was calculated as 1 g/kg using the body surface area conversion formula [68]. This dose was designated as the medium-dose (MD) group. The low-dose (LD) and high-dose (HD) groups were set at 0.5 times and 2 times of this value, i.e., 0.5 g/kg and 2 g/kg each day, respectively. The central fatigue animal model was established using a modified multiple platform method (MMPM) combined with alternate-day fasting (ADF). The detailed procedures and underlying rationale for the modeling protocol are described in our previous study [30]. Gavage administration was initiated on the 15th day of the modeling period and continued daily for 7 days. Throughout the experiment period, any animals that died during modeling, behavioral testing, anesthesia, or any other reasons were excluded from the experimental observations. Figure 14 shows a schematic overview of the experimental procedure.

### 4.4. Behavioral Testing

#### 4.4.1. Tail Suspension Test

The tail suspension test evaluates physical strength, fatigue, and motivational changes in rats under stress, reflecting both psychological and physical fatigue. On day 22, the tails of the rats were threaded through a 1.5 cm-diameter aperture in a partition, with the upper tail base secured to hang inverted for 6 min. Immobility time and struggling frequency were recorded during the final 4 min.

#### 4.4.2. Morris Water Maze

The Morris water maze test assesses spatial learning and memory in animals, delineating spatial recognition. A 1 m black PVC pool, divided into four quadrants with a central platform, was used (Zhongshi Di Development Co., Ltd., Beijing, China). The 5-day protocol included a 4-day learning phase with daily varied entry points and a 1-day probe trial without the platform. On day 22, rats were placed in the third quadrant for 120 s, with their movements recorded and analyzed for escape latency, platform crossings, and target quadrant time.

#### 4.4.3. Open Field Test

The open field test was employed on day 22 to assess rats’ spontaneous exploration and anxiety in an unfamiliar setting. Rats were placed in a 100 cm × 100 cm × 40 cm open field box (Zhongshi Di Development Co., Ltd., Beijing, China) under a HD camera for continuous 5 min recording. Their behaviors, including rearing and grooming time, were observed, while the animal motion tracking system (Noldus, Wageningen, Netherlands) analyzed total distance and central percentage [69].

#### 4.4.4. Elevated Plus Maze Test

The elevated plus maze test was employed to assess anxiety-like behaviors in rats on the 23rd experimental day. Rats were positioned head-first towards an open arm on the elevated plus maze (100 cm × 100 cm × 40 cm, Zhongshi Di Development Co., Ltd., Beijing, China), with the apparatus under continuous high-definition recording. Their behavior was monitored for 5 min, and the animal motion tracking system quantified the proportions of entries and time spent in open arms [70].

#### 4.4.5. Grip Strength Test

On the 23rd day, rat limb strength was measured using a grip strength meter (Zhongshi Di Development Co., Ltd., Beijing, China). Rats were placed on the grip plate, and their tails were pulled backward with a steady force until they let go of the plate’s outer bar. The peak grip strength of each rat was measured across three consecutive trials, and the mean of these values was determined for statistical analysis [71].

#### 4.4.6. Forced Exhaustive Swimming Test

On the 23rd day, the rats’ exercise endurance and fatigue were assessed via forced exhaustive swimming test. A lead ring weighing approximately 10% of each rat’s body weight was affixed to the tail at a point located between 1/3 and 2/3 of the tail length from the base [72,73]. The rats were gently held by the back and lowered head-first into a swimming tank (Neuroimmunology Laboratory, Beijing University of Chinese Medicine), where they swam under the burden of the added weight. Timing commenced immediately upon water immersion, and exhaustion was recorded when a rat failed to return to the surface within a 10 s period following submersion.

### 4.5. Histopathological Staining

Rats were anesthetized via intraperitoneal injection of 3% sodium pentobarbital and subsequently perfused transcardially with 4% paraformaldehyde. Brains were fixed in 4% paraformaldehyde (Servicebio, Wuhan, China) for more than 24 h, followed by paraffin embedding. Sections were stained with hematoxylin and eosin (Baiqiandu Biological Technology, Ltd., Wuhan, China), dewaxed, hydrated through a gradient alcohol series, and stained for nuclear (hematoxylin) and cytoplasmic (eosin) components. Following dehydration and xylene clearing, sections were mounted with neutral gum. Hippocampal pathology in control and model groups was assessed using a light microscope (Nikon, Tokyo, Japan). Additionally, brain sections were stained with Nissl stain after dewaxing and hydration. Sections were stained with cresyl violet (Baiqiandu Biological Technology, Ltd.,Wuhan, China), dehydrated, cleared, and mounted, and CA1 hippocampal region pathology was examined microscopically.

### 4.6. Serum Biochemical Index Detection

Following a 12 h fast, rats were euthanized with 3% sodium pentobarbital (3 mL/kg, Sigma-Aldrich, St. Louis, MO, USA) according to ethical guidelines, and 3 mL of blood were collected from the abdominal aorta using tubes without anticoagulant. After 2 h of clotting, serum was isolated via centrifugation at 3000 × *g* for 10 min at 4 °C. Serum ALT, AST, BUN, LAC, LDH, CK, and GLU concentrations were analyzed using an automated biochemical analyzer (Beckman Coulter, Brea, CA, USA).

### 4.7. ELISA

ELISA was employed to quantify glycogen, 5-HT, and DA levels. MG and LG were determined using the Muscle Glycogen Assay Kit (Cat #MB-7562A) and the Liver Glycogen Assay Kit (Cat # MB-22066A), respectively. Hippocampal 5-HT and DA contents were assessed using the 5-HT Assay Kit (Cat #MB-1983A) and the DA Assay Kit (Cat #MB-1896A). All ELISA measurements were performed using kits from Jiangsu Meibiao Biotechnology Co., Ltd. (Yancheng, China), following the manufacturer’s recommended procedures.

### 4.8. Measurement of SOD, MDA and GSH-Px

To assess oxidative stress damage in fatigued rats, the levels of SOD, MDA, and GSH-Px in hippocampal tissues were measured using specific assay kits. The Total SOD assay kit with WST-8 (Cat #S0101S, Beyotime Institute of Biotechnology, Shanghai, China) was employed to quantify SOD levels, by mixing supernatants after centrifugation with reaction reagents and measuring absorbance at 450 nm. MDA levels were quantified using a lipid peroxidation MDA assay kit (Cat #S0131S, Beyotime Institute of Biotechnology, Shanghai, China), and absorbance was measured at 532 nm. GSH-Px activity was detected with the cellular GSH-Px assay kit (Cat # S0057S, Beyotime Institute of Biotechnology, Shanghai, China), using a DTNB-based method and measuring absorbance at 412 nm. Protein concentrations were measured with the Enhanced BCA Protein Assay Kit (KeyGen Biotech, Nanjing, China), and enzyme activities were expressed as units per milligram of protein.

### 4.9. Western Blot

Hippocampal tissue was minced and homogenized in RIPA lysis buffer (P0013, Beyotime, Shanghai, China) containing protease inhibitor (P1005, Beyotime, Shanghai, China). The homogenates were incubated on ice for 30 min to ensure full lysis and subsequently centrifuged to obtain the supernatant for subsequent protein quantification. Protein concentrations were quantified with a BCA assay kit (KGP902, Keygen Biotech, Nanjing, China) according to the manufacturer’s protocol. For Western Blot, protein samples (50 μg per lane) were mixed with loading buffer, denatured by boiling at 100 °C for 10 min, and separated on a 10% SDS-PAGE gel under constant voltage. The separated proteins were then transferred to a PVDF membrane, which was subsequently blocked with 5% BSA in TBST for 2 h at 25 °C. After washing with TBST, the membranes were incubated overnight at 4 °C with the following primary antibodies: phospho-PI3K p85 (1:1000, Abcam, Cambridge, UK), phospho-AKT1 (1:1000, Wanlei, Shenyang, China), and GAPDH (1:50000, Proteintech, Wuhan, China). On the following day, membranes were incubated for 2 h at room temperature with goat anti-rabbit secondary antibodies (1:10000, Proteintech, Wuhan, China) under gentle shaking. Protein bands were visualized using enhanced chemiluminescence (ECL) reagent and imaged with the SHST imaging system (SH-Compact 523, Shenhua Technology Co., Hangzhou, China). Analysis was performed with Image J (v 1.54g) software, and the relative expression level of the target proteins was quantified by the grayscale ratio of the respective protein bands normalized to the internal control (GAPDH).

### 4.10. Serum Metabolomic Analysis

#### 4.10.1. Preparation of Samples

Blood from the rat abdominal aorta was collected and centrifuged to obtain serum, and LC-MS samples were prepared as follows: the serum sample was thawed at 4 °C and subsequently vortexed for one minute to achieve homogeneity after thawing. An appropriate amount of the thawed sample was then accurately transferred into a 2 mL centrifuge tube. Subsequently, 400 μL of methanol was added to the mixture, which was then vortexed for an additional minute. Following this, the sample was centrifuged at 12,000 rpm for 10 min at 4 °C. The supernatant was carefully harvested and transferred to a fresh 2 mL microcentrifuge tube for concentration and drying. To facilitate re-dissolution, 150 µL of a 2-chloro-l-phenylalanine solution (4 ppm) in 80% methanol-water was added to the dried residue. The supernatant was then filtered using a 0.22 μm membrane and transferred to the detection bottle for LC-MS analysis [74].

#### 4.10.2. Acquisition of LC-MS Data

The liquid chromatography analysis was conducted on a Vanquish UHPLC System (Thermo Fisher Scientific, MA, USA). Chromatographic separation was achieved using an ACQUITY UPLC^®^ HSS T3 column (2.1 × 100 mm, 1.8 µm) (Waters, Milford, MA, USA) maintained at 40 °C. The flow rate was set at 0.3 mL/min, and the injection volume was 2 μL. For LC-ESI (+)-MS analysis, the mobile phases were composed of (B2) 0.1% formic acid in acetonitrile (*v*/*v*) and (A2) 0.1% formic acid in water (*v*/*v*). The separation was carried out using a gradient elution protocol: 0–1 min, 8% B2; 1–8 min, 8–98% B2; 8–10 min, 98% B2; 10–10.1 min, 98–8% B2; 10.1–12 min, 8% B2. For LC-ESI (-)-MS analysis, the mobile phases were (B3) acetonitrile and (A3) 5 mM ammonium formate, with the same gradient profile applied. Metabolite detection was performed using a Q Exactive Focus mass spectrometer (Thermo Fisher Scientific, MA, USA) equipped with an ESI ion source. Data acquisition was carried out in Full MS-ddMS2 mode, enabling simultaneous MS1 and MS/MS scanning. The key parameters were set as follows: sheath gas pressure, 40 arb; auxiliary gas flow rate, 10 arb; spray voltage, 3.50 kV (positive mode) and −2.50 kV (negative mode); capillary temperature, 325 °C; MS1 scan range, *m*/*z* 100–1000; MS1 resolving power, 70,000 FWHM; number of data-dependent MS/MS scans per cycle, 3; MS/MS resolving power, 17,500 FWHM; normalized collision energy, 30 eV; and dynamic exclusion, automatic.

#### 4.10.3. Data Processing and Analysis

The LC-MS raw data were transformed to the mzXML format using msConvert (ProteoWizard). XCMS (v 3.12.0) software was employed to perform peak alignment, retention time adjustment, and peak area quantification [75]. Both PCA and OPLS-DA were performed using the “ropls” package for R software [76]. Metabolites satisfying the criteria of variable importance in projection score > 1 and *p* < 0.05 were identified as potential biomarkers [77]. All of the biomarkers were identified by the Human Metabolome database (URL http://www.hmdb.ca/, accessed on 20 April 2024), MassBank of North America database (URL https://mona.fiehnlab.ucdavis.edu/, accessed on 20 April 2024), MassBank database (URL https://massbank.eu/MassBank/, accessed on 20 April 2024), Metabolite Link database (URL https://metlin.scripps.edu, accessed on 20 April 2024) and NIST database (URL https://chemdata.nist.gov/, accessed on 20 April 2024). The “PMCMRplus” package (v 1.9.6) for R software (v 4.0.3) was utilized to assess the statistical differences in data among three groups [78]. An ANOVA test was applied for the comparison among the three groups. Post hoc comparisons were performed by the Dunn’s post-test with Benjamini–Hochberg (BH) correction [79]. Subsequently, the “clusterProfiler” package (v 4.6.0) for R software and MBROLE platform (URL https://csbg.cnb.csic.es/mbrole2/index.php, accessed on 25 April 2024) were employed for conducting KEGG pathway enrichment analysis of the potential biomarkers [80]. MBROLE platform was also used to find the protein interactions of the metabolites [81].

### 4.11. Statistical Analyses

Statistical analyses were performed using SPSS 26.0 (IBM, Armonk, NY, USA), with graphics created using GraphPad Prism 9.5.0 (GraphPad, San Diego, CA, USA). Any rats that died during the modeling period, behavioral testing, anesthesia, or due to any other reasons prior to the completion of the experimental protocol were excluded from all subsequent analyses. Data are expressed as mean ± SD. For normally distributed data, one-way ANOVA was applied for intergroup comparisons, followed by either the LSD or Tamhane test for post hoc pairwise analysis. In cases of non-normally distributed data, the nonparametric rank sum test was employed. A *p*-value < 0.05 denoted a statistically significant difference between the values. The group allocation was necessarily known to the individual who performed the randomization and administered the daily treatments by gavage. However, stringent blinding procedures were implemented for all outcome assessments and data analysis to prevent bias. All behavioral assessments, histopathological examinations, electron microscopy evaluations, and biochemical assays were performed by investigators blinded to the experimental conditions.

## 5. Conclusions

This study elucidates the therapeutic effects of TFK on central fatigue-related syndromes, including anxiety, depression, and cognitive impairment. Integrated network pharmacology and experimental validation revealed that the underlying mechanism is associated with the activation of the PI3K-AKT signaling pathway, which alleviates hippocampal oxidative stress and neuronal death. Simultaneously, based on metabolomics analysis, TFK can also regulate amino acid metabolism, particularly by modulating the levels of L-tryptophan and leucine in peripheral blood, thereby indirectly influencing the balance of DA and 5-HT in the brain and ultimately exerting therapeutic effects on central fatigue. This study establishes a theoretical basis for the development of TFK-derived anti-fatigue TCM and health products.

## Data Availability

The original contributions presented in this study are included in the article/supplementary material. Further inquiries can be directed to the corresponding authors.

## Supplementary Materials

The following supporting information can be downloaded at: https://www.mdpi.com/article/10.3390/ph18101545/s1, Supplementary Table S1: compounds identified in the positive ion mode; Supplementary Table S2: compounds identified in the negative ion mode; Supplementary Table S3: 30 active compounds of TFK and their network parameters; Supplementary Table S4: 482 common targets of TFK and central fatigue; Supplementary Table S5: The results of GO enrichment; Supplementary Table S6: The results of KEGG enrichment; Supplementary Table S7: active compounds and their Mol ID.
